# MUC16 promotes EOC proliferation by regulating GLUT1 expression

**DOI:** 10.1111/jcmm.16345

**Published:** 2021-02-04

**Authors:** Fang Wang, Qing Zhang, Hailing Zhang, Xiaogai Qiao, Xia Zhang, Ke Zhang, Xiaoli Gu, Lihong Wang, Jinquan Cui

**Affiliations:** ^1^ Department of Obstetrics and Gynecology The Second Affiliated Hospital of Zhengzhou University Zhengzhou China

**Keywords:** epithelial ovarian cancer, glucose transporter 1, glucose uptake, mucin 16, proliferation

## Abstract

As a common malignancy in females with a higher incidence rate, epithelial ovarian cancer (EOC) is a heterogeneous disease with complexity and diversity in histology and therapeutic response. Although great progress has been made in diagnosis and therapeutic strategies, novel therapeutic strategies are required to improve survival. Although the promoting effect of mucin 16 (MUC16) on tumour progression has been reported, the potential mechanisms remain unclear. In our study, we reported that overexpression of MUC16 was significantly related to cell proliferation and disease progression in EOC. Results from clinical specimen analysis and cell experiment support this conclusion. Patients with a high MUC16 expression usually had a worse prognosis that those with a low expression. Cell proliferation ability was significantly decreased in EOC cell lines when the knockdown of MUC16. Further study shows that the function of MUC16 in cell proliferation is based on the regulation of glucose transporter 1 (GLUT1) expression. MUC16 can control glucose uptake by regulating GLUT1 in EOC cells, thereby promoting glycogen synthesis, so that tumour cells produce more energy for proliferation. This conclusion is based on two findings. First, the significant correlation between MUC16 and GLUT1 was verified by clinical specimen and TCGA data analysis. Then, alteration of MUC16 expression levels can affect the expression of GLUT1 and glucose uptake was also verified. Finally, this conclusion is further verified in vivo by tumour‐bearing mice model. To summarize, our results suggest that MUC16 promotes EOC proliferation and disease progression by regulating GLUT1 expression.

## 1 INTRODUCTION

Ovarian cancer, as one of the most threatening female malignancies in the world, is an important disease that causes women to die.^1^ Almost 240 000 cases of OC are determined every year, with some 75% of patients are diagnosed with widespread intra‐abdominal disease at a late stage.^2^ As the most common type of ovarian cancer, the patients with epithelial ovarian cancer (EOC) are always diagnosed in advanced stage disease, because of the asymptomatic or nonspecific symptoms of early‐stage EOC and the lack of biomarkers for specific screening.^3^, ^4^ The standard treatment for EOC consists of cytoreductive surgery followed by platinum‐based chemotherapy.^5^ Although the responses of the initial tumour treatment are promising, there is a recurrence rate due to chemo‐resistance.^6^ An important mechanism is the high adaptability of cancer cells to any therapeutic modality and specifically signalling pathways to promote tumour cell survival and metastasis. Therefore, investigation of molecular mechanisms of EOC is imperative to provide novel targets for treatment of EOC.^7^

Glucose transporters (GLUTs) which are used to mediate transmembrane transport of glucose into cells are the first and rate‐limiting protein in cellular glucose utilization which is a process often exacerbated in growth and proliferation of tumour cells.^8^ Glucose transporter 1 (GLUT1), encoded by solute carrier family 2(SLC2A1),^9^ is an important member of the GLUTs family, which is the major limitation of cell glucose metabolism.^10^ Tumour metabolism has gradually become a research hotspot, so that the GLUT1 is getting more and more attention because of its pivotal role in glycolysis.^11^ The abnormal expression of GLUT1 in various tumour types, which has led it to become a novel target for tumour therapy.

Mucin 16 (MUC16), also known as CA‐125, is a transmembrane member of the mucin family and is overexpressed in many types of cancer.^12^ The correlation of MUC16 abnormal expression with tumorigenesis has been reported.^13^ MUC16 was capable of binding the tumour necrosis factor–related apoptosis‐inducing ligand, thereby inhibiting apoptosis of tumour cells.^14^ It has been reported that MUC16 mediates the interaction between tumour cells and immune cells.^15^, ^16^ MUC16 is known to inhibit the formation of immune synapse in NK cells, and it inhibits the recognition of NK cells, thus enhancing immune escape.^17^ MUC16 has been proposed to be a tumour biomarker of epithelial ovarian cancer.^18^ However, the tumour‐promoting mechanism of MUC16 is not entirely clear. It is hoped that the present study may demonstrate the molecular mechanism of MUC16 promotes EOC progression and cell survival.

## 2 MATERIALS AND METHODS

### 2.1 Clinical sample selection

Fresh tissue was collected from the operating room, immediately collected in RNAlater reagent and then stored at −80°C until further RNA extraction. The paraffin section of tissue samples from EOC patients were obtained from the Pathology Department of the Second Affiliated Hospital of Zhengzhou University. This study was approved by the local clinical research ethics committee, and an informed consent was obtained from each patient and volunteer, with available follow‐up information.

### 2.2 Cell lines and cell culture

Epithelial ovarian cancer cell lines (OVCAR3, HO8910 and SKOV‐3) were purchased from American Type Culture Collection (ATCC). All cell lines were cultured in Dulbecco's modified Eagle's medium (DMEM) (Gibco) supplemented with 10% (v/v) foetal bovine serum (FBS) (Invitrogen) in 5% CO_2_ atmosphere at 37°C. pCDH‐EF1a‐MUC16 and si‐MUC16 vectors were synthesized using siRNA and transfected into EOC cells using Lipofectamine 3000 (Invitrogen). The sequence used for MUC16 knockdown is shown in Table S1.

### 2.3 Immunohistochemistry analysis

Immunohistochemical staining was performed with antibodies against human MUC16 (#ab110640, Abcam), GLUT1(#ab115730, Abcam) and KI67 (#ab15580, Abcam). The semi‐quantitative scoring system was used to assess the stain intensity and area extent. Each specimen received a score based on the intensity of stained cells (0, 1, 2 and 3, respectively, represent blank, light yellow, yellow and brown) and the extent of stained cells (0, 1, 2 and 3, respectively, represent 0%, 1%‐29%, 30%‐69% and 70%‐100%), and the immunoreactive score was derived by multiplying the extent of stained cells by the intensity score.^19^

### 2.4 Quantitative real‐time PCR (qRT‐PCR)

Total RNA was extracted from EOC cell lines and EOC tissues using TRIzol reagent (Thermo Fisher Scientific). Reverse transcription kit (Takara Biotechnology) was used for reverse transcription. StepOnePlus™ Real‐Time PCR System (Thermo Fisher Scientific) was used to perform the qRT‐PCR assay. GAPDH was used as the endogenous control to normalize RNA expression. The relative expression level of the target RNA was calculated using 2^−ΔΔCt^.^20^ The primers used for the amplification of targets are shown in Table S2.

### 2.5 Flow cytometry analysis

Cell suspension was (1 × 10^6^) filtered through a 100‐mmol/L strainer filter and washed with PBS containing 0.5% BSA, then incubation with antibodies (MUC16, #A54127‐100, Epigentek; KI67, #A53503‐050, Epigentek) for 30 minutes at 4°C in the dark. A FACSCalibur instrument was used to analyse the cells and further analyses were performed with FlowJo‐V10 software.

### 2.6 Western blotting

Cells were harvested in RIPA Lysis Buffer and were broken by ultrasonic treatment (power: 200 W; mode: work 4 seconds, stop 3 seconds, for five times) (#DH92‐IIN, LAWSON). Supernatants were collected, and protein concentrations were quantified using the Coomassie brilliant blue G250 (#C8420, Solarbio). Total protein from each sample was separated by SDS‐PAGE and then transferred to nylon membrane using transfer device. After blocked in TBS‐T containing 5% milk for 2 hours at room temperature, membranes were incubated overnight at 4°C with primary antibodies and subsequently with HRP‐conjugated secondary antibody. Antibodies against MUC16(#29623), GLUT1(#12939) and β‐actin(#3700) were purchased from CST.

### 2.7 Cell proliferation experiment

Epithelial ovarian cancer cell lines in the exponential growth phase were collected and re‐suspended subsequently. Three different methods (Cell Counting Kit 8, plate clone forming experiment and Nuclear KI67 detection) were used for detection the differentially ability of cell proliferation.

### 2.8 Animals and tumour subcutaneous implantation model

Nude mice (female, 4∼6 weeks old) were ordered from Vital River Laboratory Animal Technology Co. Ltd and housed at SPF environment. Two cell lines, OVCAR3‐MUC16‐SH(SH) and OVCAR3‐SH‐NC(NC), were subcutaneously injected into the nude mice. Nude mice in each group (n = 5) were labelled by punching their ears, respectively. Severn days after cell injections, the solid tumours were observed in all subjects. Vernier calliper and electronic weighing scale were used for monitored tumour size and body weight of mice once every 2 days. The formula of [length (mm) × width (mm)^2^]/2 was applied to calculate the tumour volume.^21^

### 2.9 TCGA data

Gene expression in EOC was screened by The Cancer Genome Atlas (TCGA) database (http://www.cancaergenome.nih.gov), and Spearman correlation analysis was performed.

### 2.10 Survival analysis

Univariate Cox regression model was used to analyse the relationship between overall survival and mRNA expression in EOC patients, and *P* < .05 was considered significant. The expression levels of the samples were grouped as high and low based on the cut‐off point.

### 2.11 Statistical analyses

The SPSS version 17.0 was used for statistical analyses. Comparisons between groups were evaluated using the Student's *t* test. The log rank (Mantel Cox) test was used to compared survival curves. Data of flow cytometry was analysed by two‐tailed, unpaired Student's *t* tests. Statistical significance between multiple groups was determined using the Bonferroni correction.

## 3 RESULTS

### 3.1 Pro‐cancer potential of MUC16 in EOC

**FIGURE 1:**
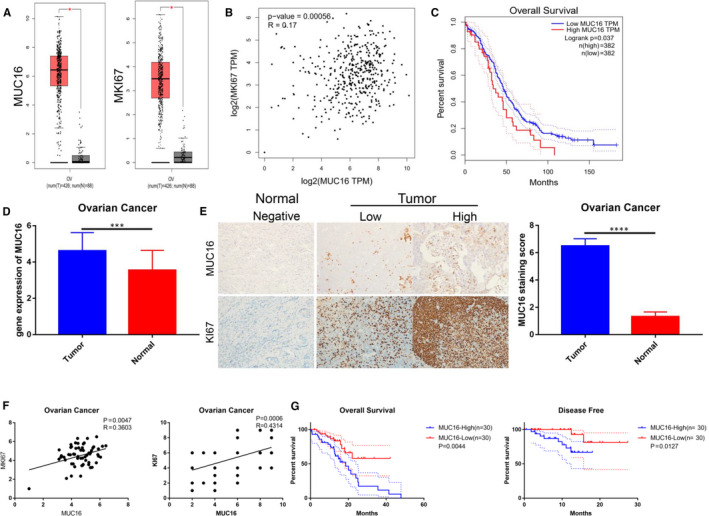
The promoting effect of MUC16 on tumour progression. A, MUC16 and MKI67 were aberrantly expressed in EOC specimens. B, The correlation of MUC16 and MKI67. C, Patients with high MUC16 expression had a significantly poorer prognosis. D, The gene expression of MUC16 in the EOC clinical specimens. E, The protein expression of MUC16 in the EOC clinical specimens(200×). F, The correlation of MUC16 and MKI67. G, MUC16 is significantly correlated with poor survival in the EOC clinical specimens (****P < .00001 and ***P < .0001)

To study the involvement of MUC16 in the regulation of EOC cell proliferation, GEPIA data were used to analyse the expression of MUC16. As shown in result, MUC16 and cell proliferation marker (MKI67)^22^ were aberrantly expressed in EOC specimens (Figure 1A). At the same time, the correlation of MUC16 and MKI67 is obvious (Figure 1B). Moreover, patients with high MUC16 expression had a significantly poorer prognosis (Figure 1C). These results suggest that the MUC16 contributes to proliferation of EOC cells and promote disease progression. Next, we validated the upregulation of MUC16 in the EOC clinical specimens at both mRNA (Figure 1D) and protein levels (Figure 1E). As expected, identical results were obtained in the clinical samples. MUC16 upregulation in EOC was observed at both gene and protein levels, and the correlation of MUC16 and MKI67 has been proved (Figure 1F). The survival analysis of MUC16 was performed in EOC samples and found that up‐regulated MUC16 is significantly correlated with poor overall survival of liver (Figure 1G). All these results were proved the fact that MUC16 is a prognostic biomarker in EOC, maybe because of promoting effect of MUC16 on tumour cell proliferation.

### 3.2 MUC16 promoting the proliferation of EOC cell lines

**FIGURE 2:**
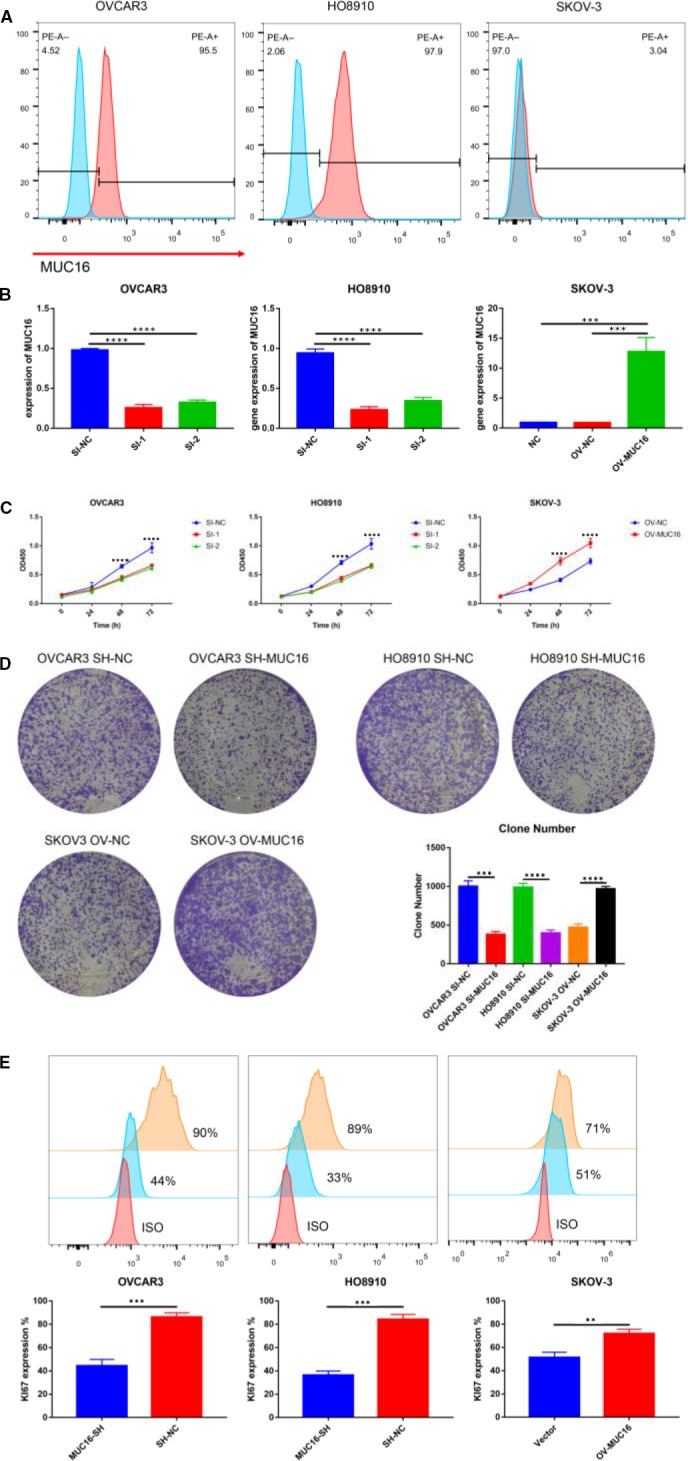
MUC16 promoting the proliferation of EOC cell lines. A, The expression of MUC16 was investigated by flow cytometry. B, Si and overexpression of MUC16 was executed for EOC cell lines. C, CCK‐8 was used for detecting proliferation of cells. D, Plate clone was used for detecting proliferation of cells. E, The expression of KI67 was investigated by flow cytometry (****P < .0001, ***P < .001 and **P < .01)

Based on the analysis of clinical specimens and publicly available data from GEPIA, we found that MUC16 expression was elevated in EOC. We explored the potential role of MUC16 using three EOC cell lines. Flow cytometry was used to detect the expression of MUC16 (Figure 2A), knockdown and overexpression of MUC16 was performed in corresponding cell lines (Figure 2B). Then, multiple methods were used to detect the proliferation of cells with different expression of MUC16 (Figure 2C‐E). As shown in result, depletion of MUC16 significantly inhibited cell proliferation, clone formation capability was also decreased by MUC16 knockdown. At the same time, expression of the proliferation marker KI67 was also strongly reduced after MUC16 knockdown. To the contrary, the opposite trend was observed after overexpression of MUC16. These results indicate that MUC16 has been implicated in regulation of cellular proliferation and is a potential tumour promoter. However, the mechanism needs further to be investigated.

### 3.3 Correlation between MUC16 and GLUT1 in EOC patients

**FIGURE 3:**
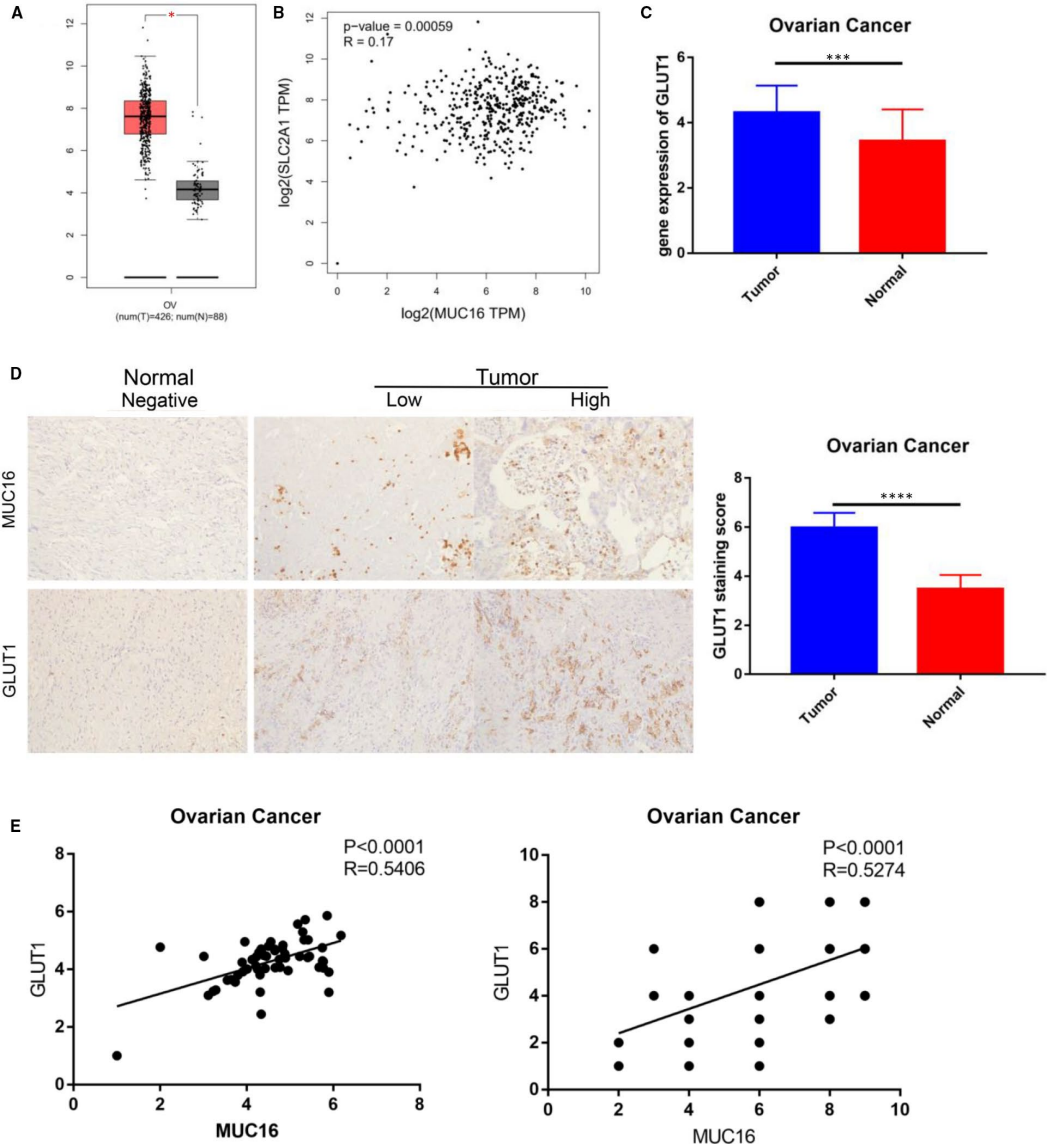
Correlation between MUC16 and GLUT1 in EOC patients. A, GLUT1 is aberrantly expressed in EOC specimens. B, The correlation of MUC16 and GLUT1. C, The gene expression of GLUT1 in the EOC clinical specimens. D, The protein expression of GLUT1 in the EOC clinical specimens (200×). E, The correlation of MUC16 and GLUT1 (****P < .00001, ***P < .0001)

Based on the above results, we look for the mechanism of MUC16 promoting tumour cell proliferation. As shown in result of GEPIA analyse, as a key protein of glucose uptake, GLUT1 is aberrantly expressed in EOC specimens (Figure 3A) and was positively correlated with MUC16 expression (Figure 3B). This was confirmed by subsequent experiments using EOC clinical specimens. GLUT1 upregulation in EOC was observed both at gene and protein levels (Figure 3C,D), and the correlation of MUC16 and GLUT1 has been proved (Figure 3E). These results suggest a potential regulatory relationship between MUC16 and GLUT1. Next, we sought to investigate whether GLUT1‐dependent glycolysis can serve as a downstream mechanism for MUC16 promoting function.

### 3.4 MUC16 promotes EOC proliferation by regulating GLUT1 expression

**FIGURE 4:**
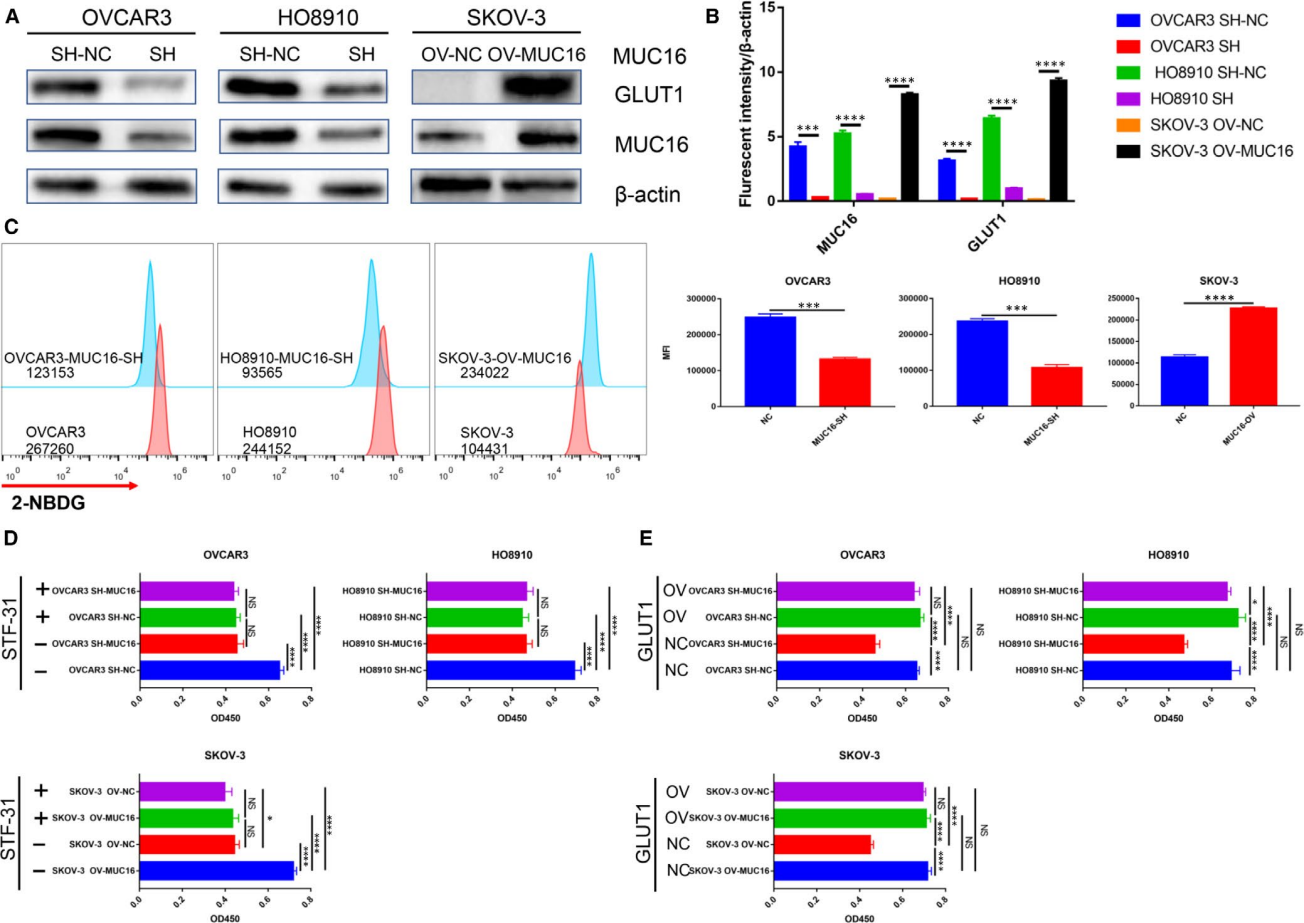
MUC16 promotes EOC proliferation by regulating GLUT1 expression. A, protein expression level of MUC16 and GLUT1 were detected by WB. B, Gray value analysis of WB result. C, 2‐NBDG uptake experiment was investigated by flow cytometry. D, GLUT1 knockdown partially blocked MUC16 induced cell proliferation. E, Increased cell viability in MUC16 overexpressing EOC cells was abolished by GLUT1 knockdown (****P < .00001, ***P < .0001, *P < .05)

To further understand the relationship between MUC16 and GLUT1 in EOC cells, different groups of cells were collected and subjected to protein extraction. For evaluated the effect of MUC16 overexpression or down‐regulation on the basal expression of GLUT1, subsequent Western blotting (WB) was executed and the result showed that altered expression of MUC16 can change the expression of GLUT1 (Figure 4A), gray value analysis revealed statistically significant differences among the groups (Figure 4B). Subsequently, 2‐NBDG (fluorescent d‐glucose analogue) was used to measure glucose uptake in EOC cell lines. As shown in this result, glucose uptake was significantly enhanced by expression of MUC16 in SKOV‐3 cells, whereas knockdown of MUC16 expression decrease glucose uptake in OVCAR3 and HO8910 cells (Figure 4C). These results suggest that MUC16 regulates the glucose uptake of EOC cells by promoting GLUT1 expression. We further investigated whether it promotes the cell proliferation through inducing GLUT1 expression, small‐molecule inhibitor and GLUT1‐overexpression cell line were used for this. We examined the effect of GLUT1 on MUC16‐induced cell proliferation and observed that GLUT1 overexpression could mimic the tumour‐promoting effects of MUC16. Meanwhile, GLUT1 knockdown blocked the MUC16‐induced cell growth in EOC cells (Figure 4D,E). The above results show that the function of MUC16 promoting cell proliferation in EOC based on expression of GLUT1.

### 3.5 MUC16 promotes EOC cell proliferation in vivo

**FIGURE 5:**
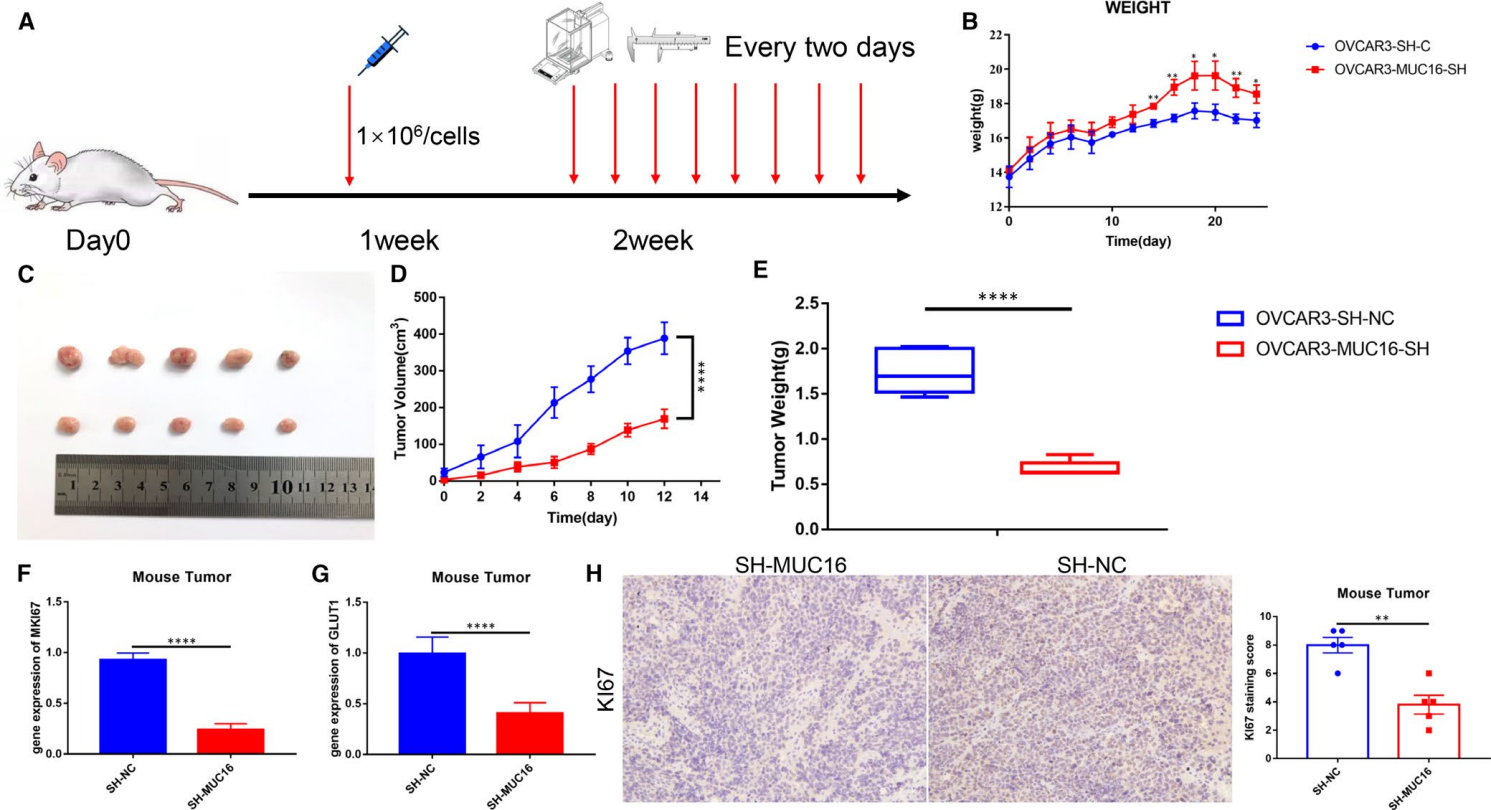
MUC16 promotes EOC cell proliferation in vivo. A, Flow chart of mouse experiment. B, Change in body weight of the mice. All mice were killed, and the stripped subcutaneous tumours were photographed (C) and weighed (D, E). The gene expression level of MKI67 (F) and GLUT1 (G) in tumour tissue was detected. H, The protein expression level of KI67 in tumour tissue was detected (200×) (****P < .00001, **P < .001 and *P < .05)

Based on the above promising in vitro results, we believe MUC16 play an important role during EOC progression, especially for tumour cell proliferation. In order to further validate this conclusion, tumour‐bearing nude mice model was established by subcutaneous inoculation with OVCAR3‐MUC16‐SH(SH) and OVCAR3‐SH‐NC(NC) and treated as described in the Materials and Methods section (Figure 5A). Mouse body weight and tumour volume were measured once every 2 days. As shown in statistical results, the weights of the mice in SH group recovered significantly compared with NC group (Figure 5B). At the same time, tumour growth curves and photograph of tumour tissues showing that tumours in the SH group grew markedly slower than those in the NC group, and the result of tumour weights measured also supporting this conclusion (Figure 5C‐E). Next, to confirm the results in vitro, total RNA was extracted from the tumours in different groups. The gene expression level of MKI67 and GLUT1 was detected with real‐time PCR. In line with our expectations, the expression of both was significantly reduced in the SH group, as compared to the NC group (Figure 5F,G). Finally, immunohistochemistry showed that the levels of KI67 decreased in the SH group, this finding is consistent with gene transcriptional level (Figure 5H). Collectively, all the above results clarified that MUC16 knockdown could inhibit the development and progression of EOC, which was achieved only by the regulatory of GLUT1 expression and consistent with its clinical relevance.

## 4 DISCUSSION

During the early stages of EOC, due to absence of typical symptoms and the lack of effective clinical screening and diagnostic techniques, the majority of EOC patients are diagnosed at late stages.^23^ Further studies will be needed to explore the pathogenesis and search new therapeutic targets. Cancer is largely dependent on metabolic reprogramming so that Weinberg introduced metabolism as a key hallmark of cancer.^24^ This characteristic of tumour cells is called ‘Warburg effect’ and it helps tumour cells produce more energy in tumour microenvironment.^25^ It is imperative that search new therapeutic targets associate with metabolic of tumour cells. Several reports indicate that high MUC16 expression is implicated in malignant metastasis, chemosensitivity and poor outcomes.^26^ Inhibiting MUC16 has been found to inhibit of tumour progression and enhance the effect of immunotherapy.^27^ José et al found that patients with MUC16 mutations had a higher TMB in all melanoma cohorts tested.^28^ However, the role of it in glycolysis of EOC is yet unclear at present. In our study, because of the abnormal expression and the correlation with disease progression and glycolysis, MUC16 is an ideal target for EOC therapy.

Available data indicate that MUC16 is a potential prognostic marker; this result is consistent with our findings. Our results show that MUC16 is more strongly expressed in EOC and that MUC16 is a pro‐tumorigenic factor. The samples with high MUC16 expression were associated with a significantly poorer prognosis for 3‐year disease‐free and overall survival than those with low expression. The overexpression of MUC16 reflects the proliferative capacity of EOC cells. All the above results are consistent with multiple published studies. Furthermore, our results further indicate the role and mechanism of MUC16 in the malignant development and glycolysis of EOC. It is well established that large amounts of glucose were needed for the rapid growth of tumour cells. As one of the most abundant glucose transporters, GLUT1 facilitates transport of glucose molecules across the cell membrane. Our results show that MUC16 is a key regulator for glycolysis, and GLUT1 is an important target of MUC16. Cell experimentation and clinical samples were used for verifying this new regulatory mechanism. Our results show that the function of MUC16 in cell proliferation is based on the regulation of GLUT1 expression. MUC16 can control glucose uptake by regulating GLUT1 in EOC cells, thereby promoting glycogen synthesis, so that tumour cells produce more energy for proliferation. At the same time, furious increases in glucose metabolite were also needed for tumour cells. And the effect of glucose metabolite on immune cells in tumour microenvironment is worth further studying, this may explain why MUC16 can affect the regulation of immune responses, which is our next‐step research direction.

It is often observed GLUT1 abnormal expression in tumour cells promote the ‘Warburg effect’.^29^ GLUT1 provide promising targets for tumour targeting therapy.^30^ In this paper, our results show that the expression of GLUT1 was significantly promoted by MUC16. Targeting MUC16 to develop anti‐tumour drugs has higher specificity than drugs target GLUT1. These results suggest that MUC16 could be a security therapeutic target in EOC. Our results provide greater variety of treatment options for EOC.

In summary, our current study showed that MUC16 promoted cell proliferation in EOC cells through promoting GLUT1‐mediated aerobic glycolysis. MUC16 promoted glycolysis, so that influenced the cell energy metabolism. However, there are still some shortcomings in the research process; further tests are needed for validation. The regulatory mechanism needs further investigation. It also remains to be proved that the influence of MUC16 on tumour therapy. In future research, we will use the CAR‐T cells therapeutic model for the study of MUC16.

## CONFLICT OF INTEREST

The authors declare that they have no competing interests.

## AUTHOR CONTRIBUTION

**Fang Wang:** Conceptualization (equal). **Qing Zhang:** Data curation (equal); Methodology (equal). **Hailing Zhang:** Data curation (equal). **Xiaogai Qiao:** Formal analysis (equal). **Xia Zhang:** Investigation (equal). **Ke Zhang:** Methodology (equal). **Xiaoli Gu:** Data curation (supporting). **Lihong Wang:** Formal analysis (equal). **Jinquan Cui:** Conceptualization (lead); Writing‐review & editing (lead).

## ETHICS APPROVAL AND CONSENT TO PARTICIPATE

The research protocol was reviewed and approved by the Ethics Committee of Zhengzhou University, and informed consent was obtained from all participants included in the study, in agreement with institutional guidelines.

## CONSENT FOR PUBLICATION

Not applicable.

## Supporting information

Table S1

Table S2

## Data Availability

The data sets used and/or analysed during the current study are available from the corresponding author on reasonable request.
