# Supplementary material for: MUC16 promotes EOC proliferation by regulating GLUT1 expression
Source: J Cell Mol Med. 2021 Feb 4;25(6):3031–40. doi: 10.1111/jcmm.16345 (PMC7957195; doi:10.1111/jcmm.16345)
Supplement: Supplementary file 1 — Table S1 [file JCMM-25-3031-s002.docx]

**Table S1.** siRNA sequence used for knockdown.

| Target Gene | Number | siRNA sequence |
| --- | --- | --- |
| MUC16 |  |  |
|  | 1 | AAGUAAAGGGCAGAGUAUGUU |
|  | 2 | AGACUAAAGUGGUAGAUGGAG |
