# Supplementary material for: MUC16 promotes EOC proliferation by regulating GLUT1 expression
Source: J Cell Mol Med. 2021 Feb 4;25(6):3031–40. doi: 10.1111/jcmm.16345 (PMC7957195; doi:10.1111/jcmm.16345)
Supplement: Supplementary file 2 — Table S2 [file JCMM-25-3031-s001.docx]

**Table S2.** Primers used for qRT-PCT.

| Gene | Forward (5'-3') | Reverse (5'-3') |
| --- | --- | --- |
| MUC16 | CCCTGAGAAATTTTGGAGTTTC | GGACTGTGTACTTCTCAGTGACTG |
| GAPDH | CTGGGCTACACTGAGCACC | AAGTGGTCGTTGAGGGCAATG |
| GLUT1 | TGAGCATCGTGGCCATCTTT | AGGCATGGAACCATTCAGGG |
| MKI67 | CTGACCCTGATGAGAGTGAGGGA | ACTCTGTAGGGTCGAGCAGG |
